# Meat Inspection: A Department of Preventive Medicine

**Published:** 1917-09-29

**Authors:** 


### MEAT INSPECTION.

### A DEPARTMENT OF PREVENTIVE MEDICINE.

The wide and penetrating reach of medical interests could hardly be more forcibly illustrated than by the course of lectures on meat inspection recently delivered at the Royal College of Physicians of London. The College, as we need hardly remind our readers, claims to be the most select and aristocratic of the medical corporations, and it is supposed to stand in a very special sense as the guardian of the more ancient and dignified of the professional traditions. Even those who openly scoff at its pretensions and brand it as an anachronism must admit its technical status, as also its capacity for survival. The stream of events may have gone past it, but the ruin, if such it be, is not without its picturesque features. With the domestic policy and internal economy of the College none but members of its household need concern themselves. But the Corporation has certain public relations, and one of these is provided by particular courses of lectures which have an annual incidence, and to which the grave and learned Fellows and Members are duly invited. The invitation is not as a rule widely accepted, but, all the same, the existence of these lecture courses is not without significance seeing that they give to the governing authorities an opportunity, by the selection of the lecturers, to show what modern movements in medicine are judged worthy of recognition.

### The Progress of Preventive Medicine.

It is here that meat inspection has its peculiar meaning in the College programme. To-day the event appears quite seemly and natural, but we cannot imagine that such a topic would have been admitted to the attention of the dignified Collegians of a previous generation. The contrast indicates the change which has come over the professional outlook and marks the progress of preventive medicine. That a medical officer of health should discourse on meat inspection to a corporation of physicians is recognised as appropriate because the business of physicians is seen to be concerned not with disease only but also with health, and the greatest triumph medicine can obtain in relation to disease is its effective prevention. In spite of its inherited conservatism even the College of Physicians cannot wholly isolate itself from the moving currents of the age, and the appearance of meat inspection on its programme is an announcement that its sleep is not quite so profound as some of its contemners would have us believe.

The lecturer on the present occasion was Dr. Wm. J. Howarth, Medical Officer of Health for the City of London, and his position obviously entitles him to speak with authority both on the existing position and on what degree of modification is required. What seems to be the most prominent fact about meat inspection as at present practised in this country is its inadequacy. The figures presented by slaughter-houses in London, where supervision is constant, afford a strong contrast to those recorded in other parts of the country. In the former about 5 per 1,000 cattle and over 2.5 per 1,000 calves and sheep are wholly condemned as unfit for human food, not to speak of portions of carcases which share the same fate. But in various urban and rural districts the maximum figures are comparatively trifling, and in not a few the medical officer of health either cheerfully announces that no unsound meat has been condemned or he makes no reference to the subject. The contrast between the City area and the other areas can have but one meaning. But this is not all. There are dodges not a few by which the unsound carcase is converted into sound money. And the trade in prepared meats offers a special opportunity. What, by its careless method of packing and transportation, seems to be boned meat intended for feeding animals does sometimes find its way into food-preparation premises, and, short of its actual discovery there, proof is difficult to obtain. Ways that are dark are not peculiar to the Heathen Chinee, and there can be no doubt that under such rules as at present exist a market is secured for meat which, by reason of the presence of disease, is not only unfit for human consumption but is full of risk to the consumer. And the fact that in recent years more instances of ill-effects to the community have been reported from prepared meat than from fresh meat carries its own message. What is to be aimed at is, of course, competent inspection of all carcases intended to be used for human food. An important step towards this end appears to be an official marking of such carcases, and this in its turn implies a common standard fixed by regulations having a universal jurisdiction and interpreted by trained and competent inspectors.

### A Local Government Board Standard.

Dr. Howarth is satisfied that at present these conditions are so far from being realised that a universal compulsory system of meat marking is impracticable. But he suggests as an improvement on the existing chaos, and as a step towards a complete method, the establishment of a standard by the Local Government Board, leaving to the districts which desire it the option to apply for permission to use the marking fixed by the Board. This arrangement would give to the central authorities the opportunity to prescribe conditions relative to the efficiency of inspection before such prayer was granted. Hence gradually, it may be hoped, the process of meat inspection in the country generally would be raised to a higher level, and an extending education might secure the position which in existing circumstances compulsion may not attempt. As to the relation of all this to the health of the community we will quote a single figure. Out of some 68,000 cattle slaughtered in the City abscess of the liver was present in 2,202, and parasitic disease of the liver in 5,969. The College of Physicians may well listen to facts of this order.

